# Highly pathogenic coronavirus N protein aggravates inflammation by MASP-2-mediated lectin complement pathway overactivation

**DOI:** 10.1038/s41392-022-01133-5

**Published:** 2022-09-14

**Authors:** Ting Gao, Lin Zhu, Hainan Liu, Xiaopeng Zhang, Tingting Wang, Yangbo Fu, Hongzhen Li, Qincai Dong, Yong Hu, Zhang Zhang, Jing Jin, Zijing Liu, Weihong Yang, Yaoning Liu, Yanwen Jin, Kaitong Li, Yongjiu Xiao, Junli Liu, Huailong Zhao, Yue Liu, Ping Li, Jibo Song, Lu Zhang, Yuwei Gao, Sisi Kang, Shoudeng Chen, Qingjun Ma, Xiuwu Bian, Wei Chen, Xuan Liu, Qing Mao, Cheng Cao

**Affiliations:** 1 Institute of Biotechnology, Academy of Military Medical Sciences, Beijing, 100850 China; 2 Institute of Physical Science and Information Technology, Anhui University, Hefei, Anhui 230601 China; 3 Beijing Key Laboratory of Bio-products Safety Assessment, Joinn Laboratories (China) Co. Ltd, Beijing, 100176 China; 4 The 940th Hospital of the People’s Liberation Army, Lanzhou, Gansu 730050 China; 5 Department of Gastroenterology, the 960th Hospital of the People’s Liberation Army, Zibo, Shandong 255300 China; 6 Academy of Military Medical Science of PLA, 666 Liuyingxi St, Changchun, 130122 China; 7 Molecular Imaging Center, Guangdong Provincial Key Laboratory of Biomedical Imaging, The Fifth Affiliated Hospital, Sun Yat-sen University, Zhuhai, 519000 China; 8 First Affiliated Hospital, Army Medical University, Chongqing, 400038 China

**Keywords:** Target identification, Infectious diseases, Infectious diseases

## Abstract

Excessive inflammatory responses contribute to the pathogenesis and lethality of highly pathogenic human coronaviruses, but the underlying mechanism remains unclear. In this study, the N proteins of highly pathogenic human coronaviruses, including severe acute respiratory syndrome coronavirus (SARS-CoV), middle east respiratory syndrome coronavirus (MERS-CoV) and severe acute respiratory syndrome coronavirus 2 (SARS-CoV-2), were found to bind MASP-2, a key serine protease in the lectin pathway of complement activation, resulting in excessive complement activation by potentiating MBL-dependent MASP-2 activation, and the deposition of MASP-2, C4b, activated C3 and C5b-9. Aggravated inflammatory lung injury was observed in mice infected with adenovirus expressing the N protein. Complement hyperactivation was also observed in SARS-CoV-2-infected patients. Either blocking the N protein:MASP-2 interaction, MASP-2 depletion or suppressing complement activation can significantly alleviate N protein-induced complement hyperactivation and lung injury in vitro and in vivo. Altogether, these data suggested that complement suppression may represent a novel therapeutic approach for pneumonia induced by these highly pathogenic coronaviruses.

## Introduction

Highly pathogenic human coronaviruses, including severe acute respiratory syndrome coronavirus (SARS-CoV), Middle East respiratory syndrome coronavirus (MERS-CoV), and SARS-CoV-2, cause severe atypical pneumonia and hyperinflammation-related syndromes.^1–4^ As of June 6, 2022, SARS-CoV-2, which causes COVID-19, has infected more than 529 million people, with a mortality rate of 1.19% (https://covid19.who.int/). Although the pathogenesis of these diseases is being intensively investigated, the mechanism by which infections cause overactivated inflammatory responses and severe respiratory failure remains unclear.

The nucleocapsid (N) protein of SARS-CoV, a 46-kDa viral RNA-binding protein,^5^ shares 91, 51, and ~30% homology with those of SARS-CoV-2, MERS-CoV and other β-coronaviruses, respectively.^3,6,7^ As one of the most abundant viral structural proteins, SARS-CoV N protein can also be detected in serum as early as 1 day after the onset of symptoms and has been used as an early diagnostic marker.^8^

The complement system functions as an immune surveillance system that rapidly responds to infection and can be activated via the classical pathway (CP), the lectin pathway (LP), or the alternative pathway (AP).^9^ In the LP, mannan-binding lectin (MBL) (or ficolins) binds carbohydrate arrays of mannan and N-acetylglucosamine residues on the surfaces of pathogens or virus-infected cells, resulting in the activation of MBL-associated serine protease-2 (MASP-2). Activated MASP-2 then cleaves C4 and C2, leading to the formation of the C3 convertase (C4bC2b), and C3, the central and most abundant component of the complement system, is cleaved by C4bC2b into C3a and C3b. C3b converts C4bC2b to C5 convertase (C4bC2bC3b), which cleaves C5 to yield C5a and C5b fragments and initiates the terminal pathway.^10,11^ The N-linked glycosylation site N330 on the SARS-CoV spike (S) protein is critical for specific interactions with MBL.^12^ Dysregulated complement activation has been implicated in the development of acute lung diseases induced by highly pathogenic viruses.^13,14^ Higher levels of activated complement C3 and C4 fragments were found in SARS patients,^15,16^ and increased C5a and terminal complement complex C5b-9 were detected in MERS-CoV-infected *hDDP4*-Tg mice.^17^ In COVID-19 patients, extensive deposition of C5b-9, C4d and MASP-2 was observed in the lungs, microvascular endothelium, and kidneys.^18–20^ Ali et al. reported that the N protein of SARS-CoV-2 directly binds MASP-2.^21^ In addition, narsoplimab (OMS721), a fully humanized immunoglobulin gamma 4 (IgG4) monoclonal antibody against MASP-2 that inhibits LP functional activity, has been used successfully in the treatment of critically ill, mechanical ventilation-dependent COVID-19 patients in a phase II clinical trial.^22^ These reports indicate that the complement pathways are activated in highly pathogenic human coronavirus infection, but the detailed mechanism underlying these excessive complement activations is not understood.

In this study, we found that the N proteins of highly pathogenic coronaviruses, including SARS-CoV, MERS-CoV and SARS-CoV-2, bind MASP-2 and significantly potentiate complement activation through MASP-2 activation. The crucial roles of MASP-2-involved complement activation in viral pathogenesis may direct the development of therapies for COVID-19.

## Results

### N proteins of SARS-CoV, SARS-CoV-2, and MERS-CoV interact with MASP-2

**Fig. 1:**
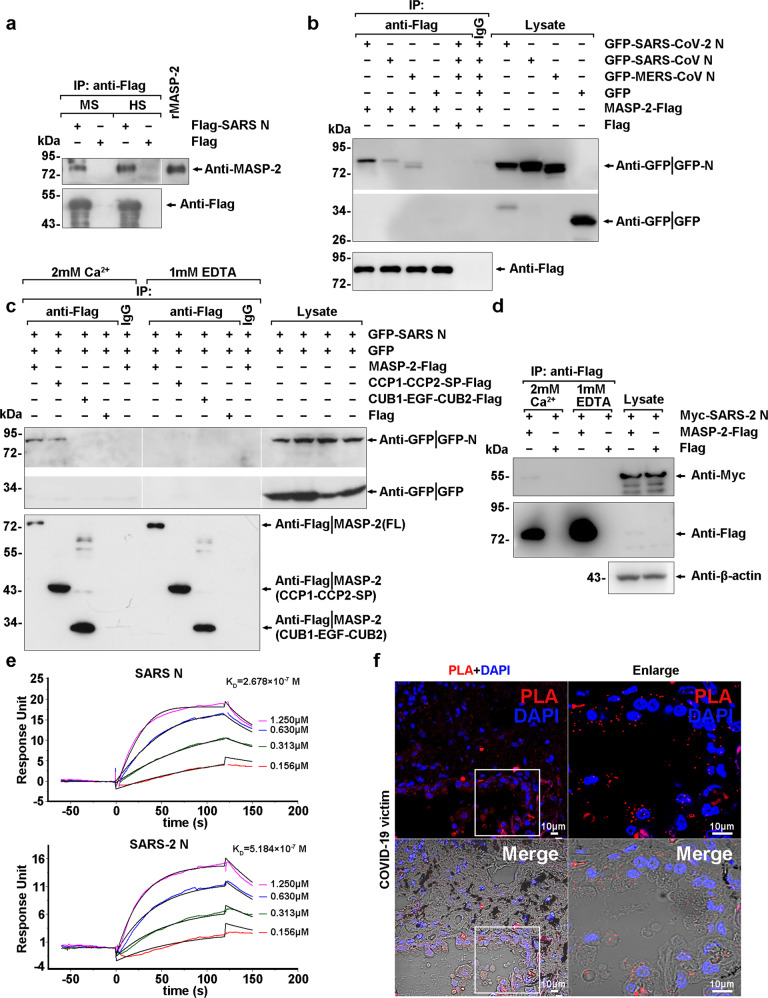
The N proteins of SARS-CoV, MERS-CoV, and SARS-CoV-2 bind MASP-2. **a** Lysates of 293T cells expressing Flag-SARS N or Flag were mixed with human serum (HS) or mouse serum (MS) and subjected to immunoprecipitation with anti-Flag beads. The absorbates were probed with the indicated antibodies. Purified recombinant MASP-2 was loaded as a marker. **b** Lysates of 293T cells expressing GFP-SARS-CoV-2 N, GFP-SARS-CoV N, or GFP-MERS-CoV N were subjected to immunoprecipitation with MASP-2-Flag-conjugated beads in the presence of CaCl_2_ and analyzed with the indicated antibodies. **c** Lysates of 293 T cells expressing GFP-SARS N and GFP were subjected to immunoprecipitation with Flag-tagged full-length (FL) MASP-2 or truncated MASP-2 (CUB1-EGF-CUB2 and CCP1-CCP2-SP)-conjugated beads in the presence of CaCl_2_ (2 mM) or EDTA (1 mM). Immunoblotting was performed with anti-GFP and anti-Flag antibodies. IgG beads and Flag beads incubated with the indicated lysates were used as negative controls. **d** Lysates of 293T cells expressing Myc-SARS-2 N were subjected to immunoprecipitation with Flag-tagged MASP-2 conjugated beads in the presence of CaCl_2_ (2 mM) or EDTA (1 mM). Immunoblotting was performed with anti-Myc and anti-Flag antibodies. Flag beads incubated with the indicated lysates were used as negative controls. Data in **a**–**d** are representative of two independent experiments. **e** SPR assays were used to detect the direct association between MASP-2 and SARS-CoV N/SARS-CoV-2 N with the indicated purified proteins. The K_D_ values were calculated using software. **f** Paraformaldehyde-fixed lung tissues from COVID-19 patients from postmortem autopsies were used for paraffin tissue sections. SARS-CoV-2 N:MASP-2 complex formation in the lung was determined by in situ PLA as indicated by the red signals. Scale bar, 10 μm

To substantiate the physical interaction between the SARS-CoV N protein and MASP-2, Flag-tagged SARS-CoV N protein (Flag-SARS N)-conjugated agarose beads were incubated with human or mouse serum, and MASP-2 in the sera was immunoprecipitated with the N protein (Fig. 1a). Similar to SARS-CoV N, exogenously expressed MERS-CoV N and SARS-CoV-2 N were both associated with MASP-2 (Fig. 1b). The association between MASP-2 and the N protein of SARS-CoV or SARS-CoV-2 was observed only in the presence of CaCl_2_ but not EDTA (Fig. 1c, d), which is consistent with the requirement for Ca^2+^ in MASP-2-MBL binding and MASP-2 autoactivation.^23^ Furthermore, the binding affinity of recombinant MASP-2 and the N protein of SARS-CoV or SARS-CoV-2 was determined by surface plasmon resonance (SPR) analysis, with K_D_ values of 2.678 × 10−7 M and 5.184 × 10^−7^ M, respectively (Fig. 1e). The SARS-CoV-2 N:MASP-2 association was also confirmed in lung tissue sections from COVID-19 victims but not cancer patients by an in situ proximity ligation assay (PLA), and the N protein:MASP-2 complexes (red fluorescent spots) were mainly distributed in pulmonary epithelial cells (Fig. 1f and Supplementary Fig. 1a). These results collectively demonstrate that the interaction between the N proteins and MASP-2 occurred in vitro and in SARS-CoV-2-infected lung tissues.

**Fig. 2:**
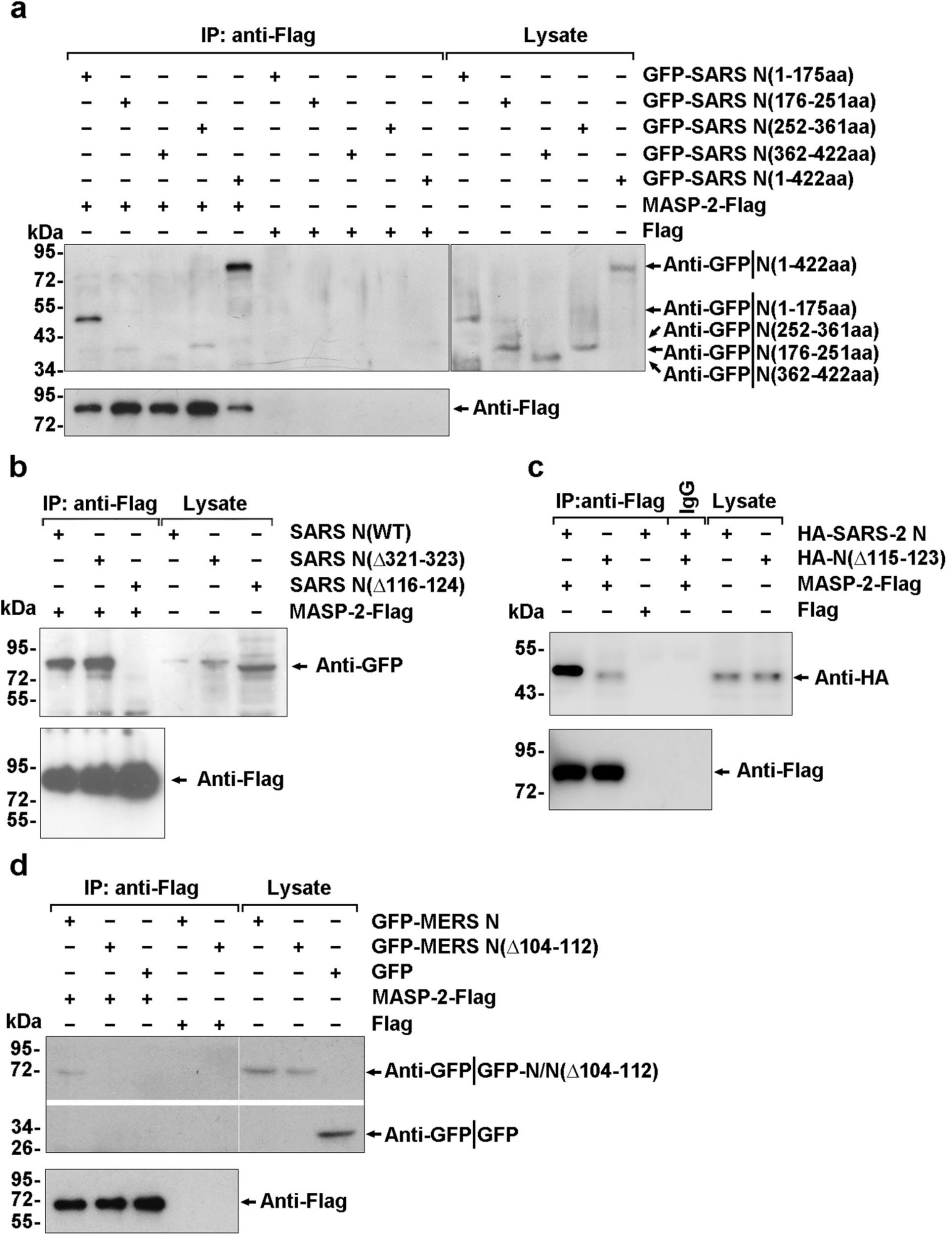
A consensus motif across the N proteins of the highly pathogenic human coronavirus TGPEAXLPY is indispensable for MASP-2 binding. **a** MASP-2-Flag-conjugated agarose beads were incubated with lysates of 293T cells expressing full-length or truncated N protein in the presence of 2 mM CaCl_2_, and the absorbates were analyzed by immunoblotting with the indicated antibodies. **b** Lysates of 293T cells expressing full-length GFP-SARS N or its mutants Δ321–323 and Δ116–124 were subjected to immunoprecipitation with MASP-2-Flag-conjugated beads in the presence of CaCl_2_ and analyzed with the indicated antibodies. **c** Lysates of 293T cells expressing HA-SARS-CoV-2 N or its truncated mutant Δ115–123 were subjected to immunoprecipitation with MASP-2-Flag-conjugated beads in the presence of CaCl_2_ and analyzed with the indicated antibodies. **d** Lysates of 293T cells expressing full-length GFP-MERS N or its truncated mutant Δ104-112 were subjected to immunoprecipitation with MASP-2-Flag-conjugated beads in the presence of CaCl_2_ and analyzed with the indicated antibodies. Data in **a**–**d** are representative of two independent experiments

Subsequently, anti-Flag immunoprecipitates of full-length or truncated Flag-MASP-2 (Supplementary Fig. 1b) were incubated with lysates of 293T cells expressing GFP-tagged SARS-CoV N protein (GFP-SARS N) or its mutants, and the adsorbates were analyzed with anti-Flag or anti-GFP antibodies by immunoblotting. Compared with other truncated regions, the CCP1-CCP2-SP region of MASP-2 (Fig. 1c) and the N-terminal domain (residues 1–175) of the N protein (Fig. 2a) were indispensable for the association.

More detailed analysis showed that amino acid residues 116–124, but not residues 321–323, which are critical for SARS-CoV N protein dimerization, were essential for the interaction with MASP-2 (Fig. 2b and Supplementary Fig. 1c). The 116–124 motif of the SARS-CoV N protein shares a high identity with the corresponding sequence in SARS-CoV-2 N (residues 115-123) and MERS-CoV N (residues 104–112) (Supplementary Fig. 1b). As expected, both the SARS-CoV-2 NΔ115-123 and MERS-CoV NΔ104-112 mutants exhibited a seriously impaired or abolished MASP-2 association (Fig. 2c, d). These results collectively demonstrate that a consensus motif (TGPEAXLPY) across the N proteins of highly pathogenic human coronavirus is conserved for MASP-2 binding.

### N proteins of highly pathogenic human coronavirus potentiate MASP-2-dependent complement activation

**Fig. 3:**
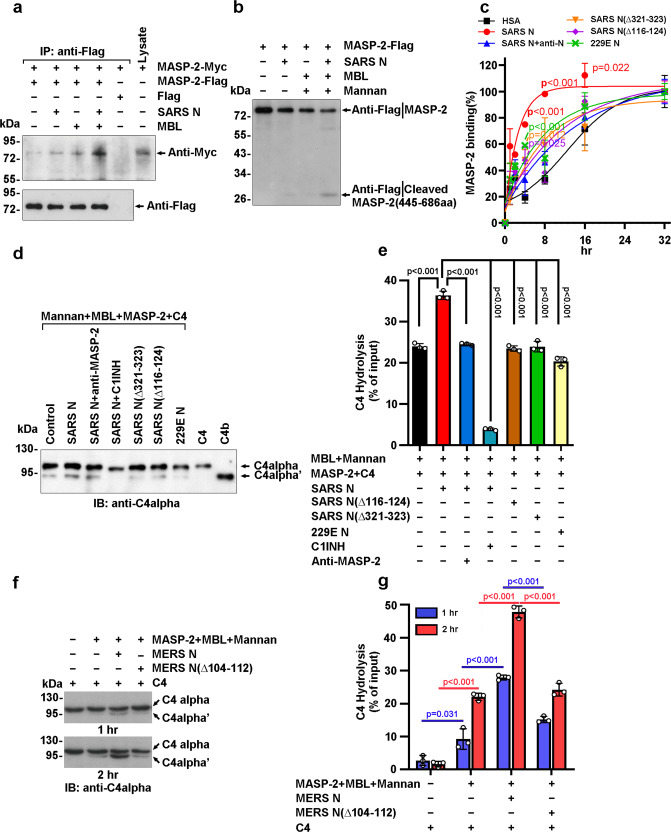
N proteins induce MASP-2 autoactivation and C4 cleavage. **a** Lysates of 293T cells expressing MASP-2-Myc were incubated with MASP-2-Flag-conjugated beads in the presence of CaCl_2,_ SARS-CoV N, and/or MBL. The adsorbents were analyzed by immunoblotting with an anti-Myc antibody. **b** Purified MASP-2-Flag was incubated with/without SARS-CoV N, MBL, and mannan at 37 °C. Cleaved MASP-2 was probed with an anti-Flag antibody. **c** Recombinant MASP-2 and N protein were diluted in buffer and incubated with preconjugated MBL in mannan-coated plates. The binding of MASP-2 to MBL in plates was detected with an anti-MASP-2 antibody. The data are presented as the mean ± SD of three tests. Statistical analysis vs. HSA was performed using unpaired two-tailed Student’s *t*-test. **d** Complement C4 was incubated with MASP-2, MBL, mannan, N proteins, C1INH, or an anti-MASP-2 antibody at 37 °C for 1 h. C4 and the cleaved truncated C4 fragment were detected with an anti-C4alpha antibody. **e** Densitometric analysis of C4 cleavage (**d**) is shown. **f** Complement C4 was incubated with/without MASP-2, MBL, mannan, MERS-CoV N protein, or mutant N protein at 37 °C for 1 h or 2 h. C4 cleavage was detected with an anti-C4alpha antibody. Data in **a**, **b**, **d**, and **f** are representative of two independent experiments. **g** The C4 cleavage rate in (**f**) was calculated by densitometric analysis with ImageJ software (*n* = 3)

The CCP1-CCP2-SP domains of MASP-2 are responsible for self-activation and substrate binding, which, in turn, mediate complement LP activation.^24–26^ To investigate MASP-2 dimerization, which charges LP activation,^27^ MASP-2-Flag-conjugated beads were incubated with lysates of cells expressing MASP-2-Myc in the presence or absence of N protein and MBL. The homodimerization of MASP-2 was potentiated by the existence of both SARS-CoV N protein and MBL (Fig. 3a and Supplementary Fig. 2a). Next, purified MASP-2-Flag was incubated with mannan and MBL with or without SARS-CoV N protein. Higher levels of the cleaved MASP-2 fragments released by MASP-2 autoactivation were detected in the presence of SARS-CoV N protein as well as MBL (Fig. 3b and Supplementary Fig. 2b).

Furthermore, the dynamics of MASP-2:MBL binding were assessed with purified MBL and MASP-2 in mannan-coated wells. Compared with human serum albumin (HSA), the binding of MASP-2 to MBL was significantly enhanced by the SARS-CoV N protein, and the potentiation was greatly abrogated by the anti-N protein antibody (Fig. 3c). Moreover, the SARS-CoV N protein bearing the Δ116–124 or Δ321–323 deletion or N protein from the less pathogenic human coronavirus 229E (HCoV-229E) showed little if any effect on MASP-2:MBL binding (Fig. 3c). These results indicate that SARS-CoV N protein-potentiated MASP-2 activation depends not only on their association but also on the dimerization of the N protein.

Next, purified C4 was incubated with MASP-2, mannan, MBL and equimolar N protein or not. C4 cleavage was significantly potentiated in the presence of SARS-CoV N protein (lanes 1 and 2 in Fig. 3d, e and Supplementary Fig. 2c) but not in the SARS-CoV N (Δ116-124 or Δ321-323) mutants or HCoV-229E N (229E N) (lanes 5–7 in Fig. 3d, e). Notably, anti-MASP-2 or anti-SARS-CoV N antibody or C1INH, an inhibitor of MASP-2,^28^ could block SARS-CoV N-potentiated C4 cleavage, suggesting that this process depends on MASP-2 activation (lanes 3 and 4 in Fig. 3d, e and Supplementary Fig. 2d, e). As expected, MERS-CoV N potentiated C4 cleavage similarly (Fig. 3f, g). These findings indicate that the N protein prompts C4 cleavage and, therefore, complement activation by MASP-2 association and activation.

**Fig. 4:**
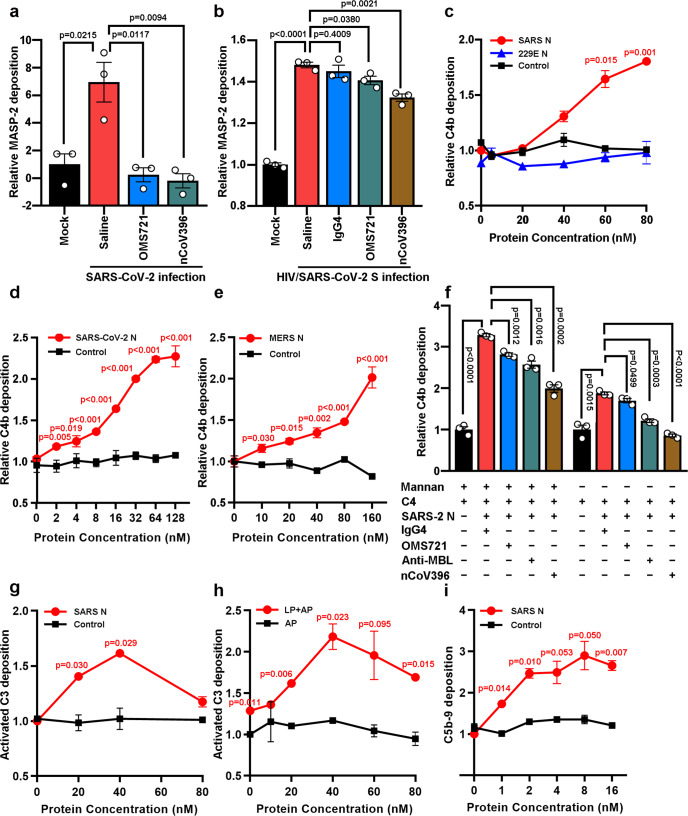
N proteins activate the lectin complement pathway via MASP-2. **a**, **b** Vero E6 cells infected with or without SARS-CoV-2 (MOI = 1) or pseudotyped virus expressing SARS-CoV-2 S (HIV/SARS-CoV-2 S, MOI = 0.2) were treated with 20% diluent serum, OMS721, nCoV396, IgG4 or SARS-CoV-2 N protein. Then, the MASP-2 deposition was analyzed. The OD450 value of non-infection was set to 1 to calibrate the relative MASP-2 deposition level. The data are presented as the mean ± SEM. **c**–**e** C4b deposition correlates with the N protein concentration of SARS-CoV, HCoV-229E, SARS-CoV-2, and MERS-CoV. The OD450 value of the control at 0 nM was set to 1 to calibrate the relative C4b deposition level. The data are presented as the mean ± SD of three tests. The statistical analysis was performed using an unpaired two-tailed Student’s *t*-test. **f** The effect of the SARS-CoV-2 N protein on C4b deposition in the presence or absence of mannan is regulated by anti-MBL antibodies, OMS721, and nCoV396. The OD450 value of SARS-2 N at 0 nM was set to 1 to calibrate the relative C4b deposition level. The data are presented as the mean ± SEM. **g** Activated C3 deposition was assessed in the presence of C1q-depleted serum (diluted in Ca^2+^-Mg^2+^ buffer) with/without N protein, and activated C3 was detected by an activated C3 antibody. **h** C1q-depleted serum was diluted in Ca^2+^-Mg^2+^ (LP+AP) or Mg^2+^-EGTA buffer (AP only) and incubated with the indicated concentration of N protein. Activated C3 was detected using an activated C3 antibody. **i** C5b-9 deposition in relation to the N protein concentration. The C5b-9 complex was detected with a C5b-9 antibody. Data in **a**–**f** are representative of three independent experiments

N-regulated complement activation was further investigated by complement deposition assays in cell-ELISA and mannan-coated microplates. Significant MASP-2 deposition was observed on the surface of the cells infected with SARS-CoV-2 or SARS-CoV-2 S protein-pseudotyped human immunodeficiency virus (HIV/SARS-CoV-2 S) with additional N protein added compared with the non-infected cells (mock) (Fig. 4a, b), and the N protein potentiated MASP-2 deposition was significantly suppressed by anti-MASP-2 or anti-SARS-CoV-2 N antibody (nCoV396^29^) (Fig. 4a, b). Accordingly, the N protein of SARS-CoV, MERS-CoV, and SARS-CoV-2, but not the HCoV-229E N protein, potentiated C4b deposition in a dose-dependent manner in C1q-depleted serum (to eliminate the classical pathway^30^) (Fig. 4c–e) but not in heat-inactivated or MBL-depleted serum (Supplementary Fig. 3a–c). Furthermore, SARS-CoV-2 N protein potentiated C4b deposition could be blocked by anti-MBL, anti-MASP-2 or anti-N protein (Fig. 4f). Much less but detectable C4b deposition was observed in the wells without mannan coating (Fig. 4f). Taken together, these results suggest that the S protein or mannan and MBL are very important for MASP-2 activation, and the activation is significantly potentiated by the N protein.

The deposition of activated C3 was also evidently increased along with the increase in SARS-CoV N protein levels up to ~40 nM (Fig. 4g), suggesting an enhanced activity of C3 convertase. In addition, the SARS-CoV N protein had little or no effect on activated C3 deposition in calcium-free buffer containing EGTA, suggesting that SARS-CoV N protein-potentiated C3 activation occurs through the LP but not the AP, in which C3 activation is Ca^2+^-independent (Fig. 4h). C3b deposition was decreased in the presence of a high concentration of N protein (Fig. 4g, h), possibly due to the further cleavage of C3b by soluble inhibitors in serum (such as factor H and factor I) when the surface was coated with high densities of C3b.^31,32^ Subsequently, as a result of amplified complement cascades, a significantly increased deposition of the C5b-9 complex was induced by SARS-CoV N protein at a much lower concentration (Fig. 4i), similar to that observed in patient sera.^8^

Activated complement plays a crucial role in the efficient phagocytosis of pathogens and cellular debris by C3b- or C5b-mediated opsonization.^33^ Complement-dependent phagocytosis of *E. coli* by mouse peritoneal macrophages was remarkably enhanced by SARS-CoV N protein but not HSA (Supplementary Fig. 3d), demonstrating that SARS-CoV N protein effectively potentiated complement-mediated opsonization.

### Complement cascade was activated by the N protein of highly pathogenic coronavirus in mice and COVID-19 patients

**Fig. 5:**
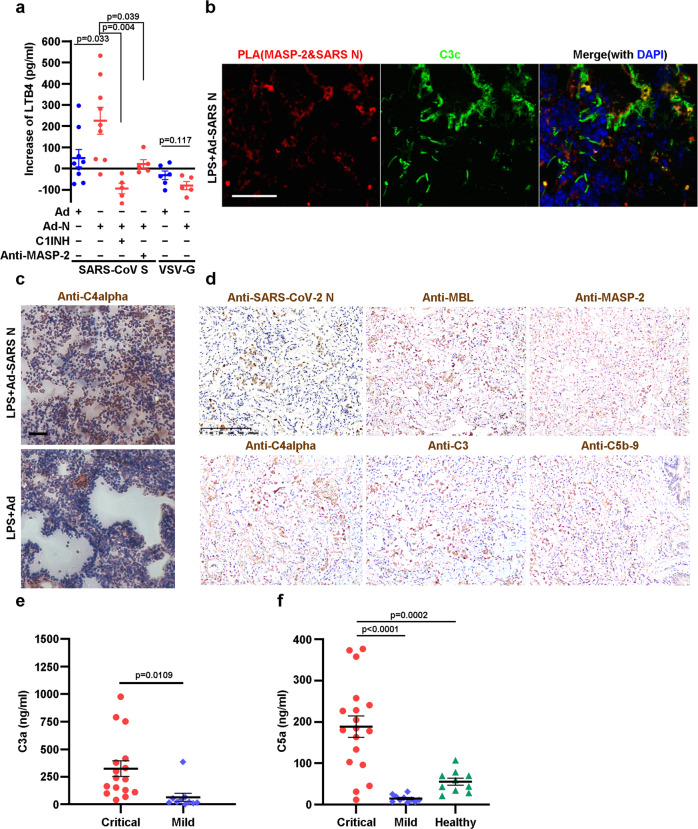
N proteins potentiate pseudovirus/LPS/SARS-CoV-2-induced pneumonia with complement activation. **a** BALB/c mice were pre-infected with Ad-SARS N or Ad (1 × 10^9^ PFU) on days 1, 2, and 3, and the SARS-CoV S protein or VSV-G pseudotyped HIV virus was injected on the 4th day via the tail vein. Sera were collected at 0 and 6 h post-pseudovirus injection, serum LTB4 was detected, and an increase in the serum LTB4 levels was observed. **b** Mice were infected with 1 × 10^8^ PFU Ad-SARS N, and LPS was injected on the 7th day post-infection. The mice were sacrificed 6 h after the LPS challenge. SARS-CoV N and MASP-2 complex formation in frozen lung sections was measured by in situ PLA (red signals). Deposited C3 fragments were stained with a FITC-labeled anti-C3c antibody (green). Scale bar, 50 μm. **c** Frozen lung sections from the indicated mice in (**b**) were stained with an anti-C4alpha antibody. Data in **a**–**c** are representative of two independent experiments. **d** Paraformaldehyde-fixed lung tissues from COVID-19 postmortem autopsies were used for paraffin tissue sections and IHC staining with the indicated antibodies. Microphotography was carried out by an Olympus BX52 microscope under a 10× objective. Scale bar, 200 μm. Serum C3a (**e**) and C5a (**f**) levels in critical COVID-19 patients were analyzed by ELISA. The data are presented as the mean ± SEM. The statistical analysis was performed using an unpaired two-tailed Student’s *t*-test

To investigate the effect of the N protein on complement-mediated inflammatory responses in vivo, mice were pre-infected with adenovirus expressing SARS-CoV N (Ad-SARS N), and the N protein was detectable in mouse serum within one week (Supplementary Fig. 4a). Then, we injected SARS-CoV spike protein-pseudotyped HIV (HIV/SARS-CoV S) to induce complement activation in vivo (Supplementary Fig. 4b, upper panel). The production of leukotriene B4 (LTB4), an arachidonic acid metabolite and a potent inflammatory factor agent induced by membrane damage, including membrane attack caused by complement activation,^34,35^ was significantly increased in the Ad-SARS N pre-infected mice compared with that in the Ad pre-infected mice, reflecting the higher inflammation level induced by N protein (Fig. 5a, lanes 1 and 2). Importantly, N protein-potentiated LTB4 production was significantly suppressed by the anti-MASP-2 antibody and C1INH (Fig. 5a, lanes 3 and 4). As a control, little, if any, induction of LTB4 production was observed following vesicular stomatitis virus G protein-pseudotyped virus (HIV/VSV-G) challenge (Fig. 5a, lanes 5 and 6), which is consistent with the findings that VSV-G did not bind MBL.^36^ N protein-mediated complement overactivation was further evaluated in an LPS-induced inflammatory mouse model in which the MASP-2-involved LP can be activated by LPS.^37^ Mice pre-infected with Ad-SARS N were challenged with LPS and sacrificed 6 h after the challenge (Supplementary Fig. 4b, lower panel). Similar to COVID-19 patient lung tissues (Fig. 1f), the in vivo association between MASP-2 and the SARS-CoV N protein was demonstrated by in situ PLA in the lungs of the Ad-SARS N pre-infected, LPS-challenged mice (Fig. 5b and Supplementary Fig. 4c), and a higher level of the cleaved MASP-2 fragment was also observed in these mice (Supplementary Fig. 4d). Immunohistochemical (IHC) analysis also demonstrated that the deposition of C4b and activated C3 staining in the lung were evidently increased in the mice expressing the SARS-CoV N protein (Fig. 5c, upper panel and Fig. 5b) compared with the weak staining observed in the control (Fig. 5c, lower panel and Supplementary Fig. 4c). These results show that the SARS-CoV N protein could promote MASP-2 and, thus, LP activation in an LPS-induced inflammatory mouse model.

Next, the paraformaldehyde-fixed lung tissue of COVID-19 victims was collected and subjected to IHC staining. Strong positive signals of the SARS-CoV-2 N protein and complement cascade components involved in LP^38^ were detected in lung tissue from COVID-19 patients (Fig. 5d). Meanwhile, significantly higher serum C3a (Fig. 5e) and C5a (Fig. 5f) levels were observed in critical, but not mild, COVID-19 patients.^39,40^ These data collectively indicate that the complement pathways could be aggressively activated in mice infected with Ad-SARS N and critical COVID-19 patients.

### N proteins aggravate LPS- and coronavirus-induced pneumonia by MASP-2-involved complement activation

**Fig. 6:**
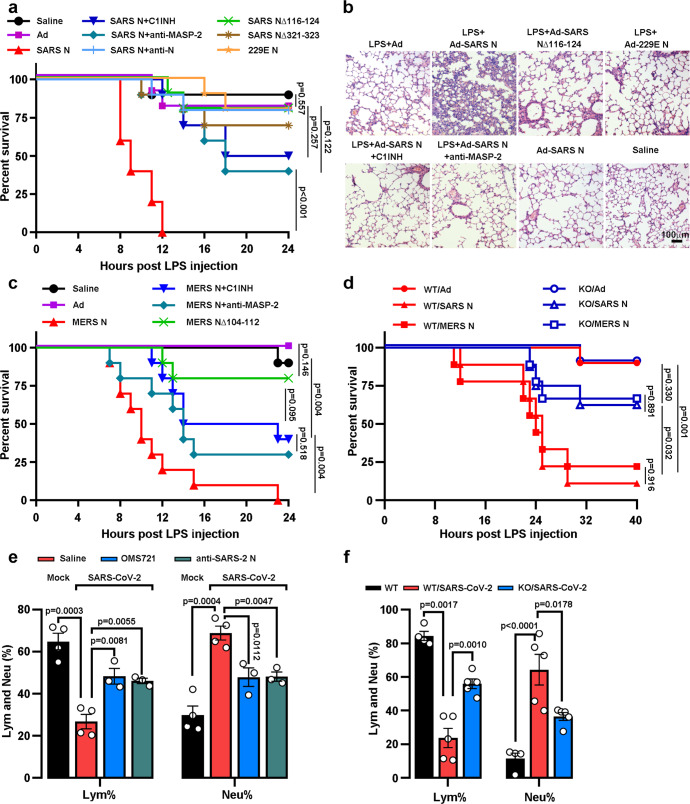
The administration of anti-MASP-2 antibodies recovered the abnormal inflammation in SARS-CoV-2-infected mice. **a** BALB/c mice (10/group) were infected three times (days 1, 2, and 3) with 1 × 10^9^ PFU Ad-SARS N, Ad or saline, and LPS was administered on the 7th day. Antibodies or C1INH were injected 30 min before LPS injection. The survival rate of the mice is presented, and the statistical analysis was performed using a log-rank test. **b** Lung paraffin sections from the indicated mice in (**a**) were analyzed by HE staining. Scale bar, 100 μm. **c** BALB/c mice were pre-infected with 1 × 10^9^ PFU Ad-MERS N or Ad and treated with LPS, an anti-MASP-2 antibody or C1INH as mentioned above. The mouse survival curves were plotted, and the statistical analysis was performed using a log-rank test. **d** *Masp2*^*−/−*^ (KO) and *Masp2*^+/+^ (WT) C57BL/6N mice were infected with N-expressing adenovirus or Ad and injected with LPS (10 mg/kg) as mentioned above. The mouse survival curves were plotted, and statistical analysis was performed using the log-rank test. **e** BALB/c mice pre-infected with 10^5^ PFU mouse-adapted SARS-CoV-2 (SARS-CoV-2 MA) were treated with saline or an anti-MASP-2 antibody (OMS721) or anti-MASP-2 antibody (nCOV396) as mentioned. Six days after infection, the lymphocyte percentage (Lym%) and neutrophil percentage (Neu%) in the mice were measured. Error bars, mean ± SEM. The statistical analysis was performed using an unpaired two-tailed Student’s *t*-test. **f** *Masp2*^+/+^ (WT) and *Masp2*^*−/−*^ (KO) C57BL/6N mice were infected with 10^5^ PFU SARS-CoV-2 MA. Six days after infection, the Lym% and Neu% in the mice were measured. Error bars, mean ± SEM. The statistical analysis was performed using an unpaired two-tailed Student’s *t*-test

Furthermore, to assess whether the N protein potentiated complement activation in pneumonia, mice pre-infected with Ad-SARS N or Ad-vector were challenged with LPS to induce pneumonia.^41^ All mice pre-infected with Ad-SARS N died within 12 h after the LPS challenge, while 8 of 10 mice pre-infected with Ad survived (Fig. 6a and Supplementary Fig. 4b, lower panel). Severe lung damage and massive inflammatory cell infiltration were observed in the dying mice (Fig. 6b). In accordance, the pre-infection with the Ad-229E N or Ad-SARS N mutants (NΔ116–124 or NΔ321–323) had far fewer effects on mortality (Fig. 6a). Accordingly, when an anti-N antibody, an anti-MASP-2 antibody or C1INH was administered simultaneously with LPS to the Ad-SARS N pre-infected mice, both the mortality and lung tissue inflammation induced by LPS were significantly improved (Fig. 6a, b). Similarly, the mice pre-infected with adenovirus expressing MERS-CoV N (Ad-MERS N), but not its Δ104-112 mutant, 100% died within 24 h after the LPS challenge, which could also be partially rescued by C1INH and the anti-MASP-2 antibody (Fig. 6c). Then, *Masp2*^*−/−*^ mice (Supplementary Fig. 5a, b) were subjected to the same treatment as shown in the lower panel of Supplementary Fig. 4b. The LPS challenge induced high mortality in wild-type mice pre-infected with Ad-SARS N or Ad-MERS N, and a significantly lower mortality was observed in the *Masp2*^*−/−*^ littermates (Fig. 6d and Supplementary Fig. 5c).

Furthermore, compared to the uninfected mice, the mice pre-infected with mouse-adapted SARS-CoV-2 (SARS-CoV-2 MA) showed a significantly decreased percentage of lymphocytes (Lym%) and an increased percentage of neutrophils (Neu%) (Fig. 6e), which are frequently observed in SARS-CoV-2-infected patients.^42^ The administration of a recombinant anti-MASP-2 antibody (OMS721) or anti-SARS-CoV-2 N antibody (nCoV396) resulted in a compromised Lym% decrease and Neu% increase compared with saline treatment (Fig. 6e). Accordingly, a significantly lower Lym% decrease and Neu% increase were observed in the *Masp2*^*−/−*^ mice compared to those observed in the wild-type littermates upon SARS-CoV-2 MA infection (Fig. 6f). Taken together, these results suggest that suppression of MASP-2-mediated complement overactivation may provide an approach for the treatment of pneumonia in pathogenic coronavirus-infected patients.

## Discussion

**Fig. 7:**
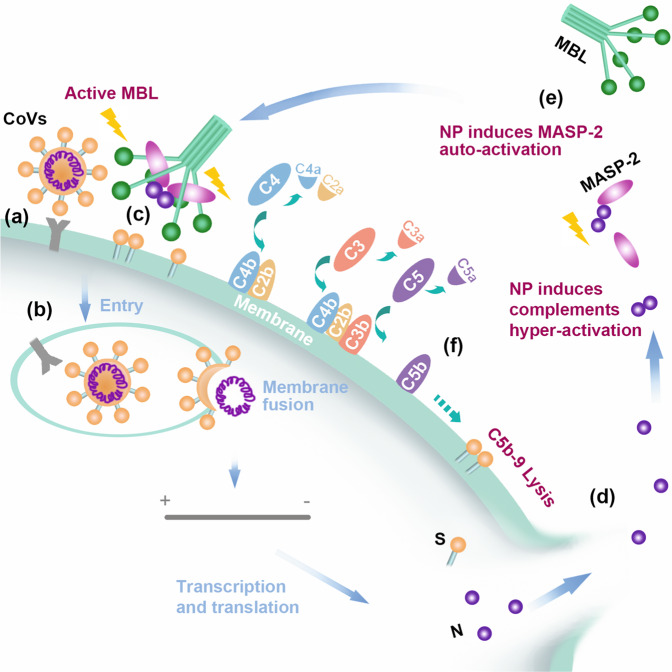
Schematic representation of the MBL pathway overactivated by the N protein of SARS-CoV, MERS-CoV, or SARS-CoV-2. **a** Virus binds the host cell surface. **b** Virus enters host cells and expresses viral proteins, including S and N proteins. **c** The S protein activates MBL. **d** N protein is released by secretion or after cell or virus lysis. **e** Extracellular soluble N protein dimers interact with MASP-2 and promote MASP-2 autoactivation and binding to MBL. **f** The accelerated activation of MASP-2 induces downstream complement cascade overactivation of the MBL pathway

In this study, the N proteins of highly pathogenic coronaviruses, including SARS-CoV N, MERS-CoV N, and SARS-CoV-2 N, were demonstrated to bind MASP-2 and potentiate the MBL-dependent activation of MASP-2, leading to hyperactivation of the LP complement cascade in vitro, in mice, and in COVID-19 patients (a schematic summary is shown in Fig. 7). Coronavirus N protein-mediated MASP-2 and thereby complement cascade overactivation can aggravate pneumonia in mice (Fig. 6).

The findings were supported by a panel of clinical and basic investigations showing that complement activation plays important roles in the pathogenesis of COVID-19, and thus, complement may serve as a driver of and therapeutic target for SARS-CoV-2.^43–45^ Both the S and N proteins of SARS-CoV-2 were observed to be directly recognized by components of the LP, leading to complement activation.^21,46^ Accordingly, the complement components involved in the LP (MBL, MASP-2, C3, and C5b-9) were increased in lung tissue from COVID-19 patients (Fig. 5d). Significantly higher levels of serum C3a and C5a were present in critical COVID-19 patients (Fig. 5e, f), which was supported by the findings that high levels of C5a and C5b-9 were present in severe COVID-19 patients, and the levels were correlated with clinical severity.^39,40^ Moreover, pre-infection with Ad-SARS N or Ad-MERS N evidently increased the fatality of LPS-induced pneumonia (Fig. 6a–c), underlining the potential role of the N protein in the inflammatory injury to tissue caused by the massive LPS released from secondary bacterial infections frequently occurring in SARS, COVID-19 and MERS patients.^1,47,48^

Our findings showed that the SARS-CoV N protein potentiated the binding of MASP-2 to MBL in mannan-coated microplate wells (Fig. 3c), and the CCP1-CCP2-SP region of MASP-2 was responsible for the interaction with SARS-CoV N (Fig. 1c). These results suggest that the N protein potentiated the MASP-2:MBL interaction by inducing conformational changes or MASP-2 activation, since the CUB1-EGF domains of MASP-2 are responsible for the MBL association.^49^ Ali et al. reported that SARS-CoV-2 N directly interacts with MASP-2 and promotes MASP-2-mediated C4 cleavage.^21^ Our results also show that the N protein induced the autoactivation of MASP-2 in vitro (Fig. 3b). These data raise the question of whether MASP-2 serves as a pattern recognition molecule (PRM). As shown in Fig. 4a, b, the N protein of SARS-CoV-2 potentiated MASP-2 deposition in SARS-CoV-2-infected cells or HIV/SARS-CoV-2 S-infected cells, but little if any effects were observed in the cells that did not express the S protein. The N protein failed to potentiate complement activation in MBL-depleted serum (Supplementary Fig. 3b, c). In addition, the SARS-CoV-2 N protein potentiated C4b deposition was also dependent on the presence of mannan, which could be blocked by anti-MBL, anti-MASP2 or anti-SARS-CoV-2 N antibodies (Fig. 4f). These findings indicate that the S protein on the cell surface (or mannan in microplate wells) and MBL are largely indispensable for MASP-2 activation and that activation is significantly potentiated by the N protein. These results suggest that the MBL-bound S protein of SARS-CoV-2 served as a PRM and that the N protein potentiated LP activation. Stravalaci et al. also suggested that SARS-CoV-2 S protein-bound MBL served as a PRM.^46^ However, the N protein of SARS-CoV-2 can still partially activate the complement pathway in the absence of mannan (Fig. 4f), suggesting that N protein-potentiated MBL-independent MASP-2 autoactivation may play a minor role in complement activation.

The lethality of LPS-exposed mice by SARS-CoV N and MERS-CoV N could be blocked by the corresponding anti-N antibody treatments but not completely by C1INH or anti-MASP-2 antibody (Fig. 6a, c), suggesting that in addition to promoting complement activation, SARS-CoV and MERS-CoV N protein may affect other pathways. Virus-specific CD8^+^ T cells can greatly ameliorate recovery from respiratory diseases such as avian influenza, SARS, and MERS and persist long-term as cross-reactive memory pools.^50–53^ In addition, B cells elicit an early response against the N protein and N-specific neutralizing antibody production at the early stage of highly pathogenic coronavirus infection.^52,54^ These studies suggest that the highly pathogenic coronavirus N protein may not only activate complement but also affect CD8^+^ T cells and antibody responses to promote inflammatory responses. However, the specific mechanism remains to be further studied.

The hyperactivation of complement was found to contribute to endothelial cell injury, thrombosis and intravascular coagulation, ultimately leading to multisystemic organ failure and excessive coagulation in COVID-19 patients.^18,55,56^ The potent anaphylatoxin C5a attracts neutrophils and monocytes to the infection site, causes tissue damage by oxidative radical formation and enzyme release, and leads to the activation of the coagulation system.^43^ These studies suggest that the coagulation-related symptoms in COVID-19 may also be associated with the abnormal activation of complement. Thus, complement cascade-targeted immunomodulation may provide new approaches against highly pathogenic coronavirus-induced inflammation, complement-related coagulopathy, and secondary bacterial infection-related diseases. Our findings provide a rationale for the clinical use of anti-MASP-2 antibodies and anti-C5a antibodies for COVID-19 treatment. Based on our primary data,^57^ narsoplimab, an anti-MASP-2 antibody also known as OMS721 developed by Omeros Corporation, was first compassionately administered to critical COVID-19 patients.^22^ The study found that all six patients requiring mechanical ventilation prior to treatment survived and were discharged from the hospital after the treatment, while substantial mortality rates of 33% and 53% were observed in the two control groups with similar entry criteria and baseline characteristics used for retrospective comparison.^22^ Rapid and sustained improvement was observed across all assessed markers of cell damage and inflammation following narsoplimab treatment, including circulating endothelial cell (CEC) counts, interleukin-6 (IL-6), IL-8, C-reactive protein (CRP) and lactate dehydrogenase (LDH).^22^ In another randomized, open label phase II clinical trial completed in the Netherlands by InflaRx (NCT04333420), an anti-C5a antibody (IFX-1, also known as vilobelimab^58^) showed potential efficacy in critical COVID-19 patients, improved lymphocytopenia, and reduced lactate dehydrogenase concentrations.^58,59^ Recently, a phase III clinical trial of vilobelimab showed a 43% reduction in 28-day all-cause mortality compared with placebo treatment in a prespecified subgroup analysis of patients in Western Europe with more severe disease (https://www.inflarx.de/Home/Investors/Press-Releases/03-2022-InflaRx-Announces-Encouraging-Phase-III-Topline-Results-from-PANAMO-Trial-of-Vilobelimab-in-Severe-COVID-19-Patients.html). These data further support that N protein-potentiated complement overactivation plays important roles in the pathogenesis of SARS-CoV-2.

Our findings have uncovered the mechanism underlying the excessive complement activation induced by highly pathogenic coronavirus infection, which has largely contributed to aggravated inflammatory lung injury in severe COVID-19 patients. Either blocking the N protein:MASP-2 interaction or suppressing complement activation can significantly alleviate N protein-induced complement hyperactivation and lung injury in vitro and in vivo. Complement suppression may represent a novel therapeutic approach for pneumonia induced by these highly pathogenic coronaviruses.

## Materials and methods

### Ethics statement

The study was performed with the approval of the ethics committee of the Beijing Institute of Biotechnology, Beijing, China, and conformed to the relevant regulatory standards. All animal studies were completed in the experimental animal center of the Academy of Military Medical Sciences, China (license number: SCXK-(Army) 2007-004, licensed by the Ministry of Science and Technology of China).

Study with sera or tissue samples from COVID-19 patients or victims were carried out under the approval of the hospital ethics committee (Ethics Approval of Huoshenshan hospital in Wuhan, Approval No. HSSLL006). The informed consents were obtained from the family member of the victims for autopsy, and were waivered for the use of the remaining sera from the clinical laboratory of the hospital in the emergency situations.

### Cell culture and transfections

The 293T cell line was obtained from the Cell Resource Center of Peking Union Medical College. The cells were grown in Dulbecco’s modified Eagle’s medium (DMEM, GIBCO) supplemented with 10% heat-inactivated fetal bovine serum (FBS, HyClone), 2 mM L-glutamine, 100 units/ml penicillin, and 100 µg/ml streptomycin. The cells were transfected with plasmid DNA using Lipofectamine 3000 (Invitrogen) according to the manufacturer’s protocol.

### Vectors and epitope tagging of proteins

The N gene of SARS-CoV (GenBank Accession: AY274119) was amplified by RT-PCR from SARS-CoV RNA from patient serum samples (upstream primer: 5′-CGGAATTCCATATGTCTGATAATGGACCCCAA-3′; downstream primer: 5′-CGGGATCCTTATGCCTGAGTTGAATCAGC-3′) and cloned into the pcDNA3-based Flag vector (Invitrogen), pCMV-Myc (Clontech), pGEX-4T-2 (GE Healthcare), and the BglII and EcoRI sites of pEGFPC1 (Clontech). The N gene of MERS-CoV (GenBank Accession: NC_019843) was chemically synthesized (HXRK Co., Ltd.) and cloned into the pcDNA3-based Flag vector at the BamHI and EcoRI sites. The N gene of SARS-CoV-2 was chemically synthesized (General Biosystems (Anhui) Co., Ltd.) and cloned into the pcDNA3.1-based HA vector at the KpnI and XbaI sites. The *MASP2* gene was amplified from a human hepatocyte cDNA library and inserted into corresponding vectors.

### Immunoprecipitation and immunoblot analysis

Cell lysates were prepared in lysis buffer (50 mM Tris-HCl (pH 7.5), 150 mM NaCl, and 1% Nonidet P-40) containing 1× protease inhibitors (cOmplete™ EDTA-free Protease Inhibitor Cocktail, Roche) with 2 mM CaCl_2_ or 1 mM EDTA. Soluble proteins were subjected to immunoprecipitation with anti-Flag M2 agarose (Sigma). Then, the adsorbates were separated by SDS-PAGE and transferred onto an Immobilon-P transfer membrane (Millipore) by semi-dry transblot (Bio-Rad). The membrane was blocked with 5% Western-Blocker (Bio-Rad). Immunoblot analysis was performed with horseradish peroxidase (HRP)-conjugated anti-Flag (Sigma), anti-β-actin (Sigma), anti-green fluorescent protein (GFP) (Clontech), anti-MASP-2 (Santa Cruz), anti-C4α (Santa Cruz), HRP-conjugated anti-Myc (Santa Cruz), and goat anti-mouse immunoglobulin G (IgG) (Amersham/Pharmacia) antibodies. The antigen-antibody complexes were visualized by chemiluminescence (GE Healthcare).

### Purification of SARS-CoV and MERS-CoV N protein

As previously described,^60^ pET22b-SARS/MERS-CoV N was transformed into the *E. coli* expression strain BL21 (DE3). After induction with 1 mM IPTG for 8 h, the bacteria were harvested by centrifugation and resuspended in buffer A (25 mM Na_2_HPO_4_/NaH_2_PO_4_ (pH 8.0), 1 mM EDTA, and 1 mM DTT) before sonication. Soluble N protein in the lysate was purified with ion-exchange chromatography with SP-Sepharose Fast Flow (25 mM Na_2_HPO_4_/NaH_2_PO_4_ (pH 8.0), 1 mM EDTA, 1 mM DTT, and 0.35–0.5 M NaCl), followed by Superdex 200 gel filtration (GE Healthcare) and elution with buffer A. *E. coli* transformed with the vector pET22b was lysed as described above, and the eluate was used as a negative control for the purified N protein. Purified SARS-CoV-2 N-His was obtained from General Biosystems (Anhui) Co. Ltd.

### Purification and renaturation of MASP-2

Recombinant protein expression and renaturation were performed as previously described.^61,62^ In brief, pET22b-MASP-2 was transformed into the expression strain BL21 (DE3). After induction with 1 mM IPTG, the cells were harvested and sonicated. The inclusion bodies were solubilized in 6 M GuHCl, 0.1 M Tris-HCl (pH 8.3), and 100 mM DTT at room temperature; then, the solubilized proteins were diluted into refolding buffer containing 50 mM Tris-HCl, 3 mM reduced glutathione (Sigma), 1 mM oxidized glutathione (Sigma), 5 mM EDTA, and 0.5 M arginine at 4 °C and incubated for 24 h. The renatured protein was dialyzed against 20 mM Tris-HCl pH 7.4 and 140 mM NaCl at 4 °C, concentrated with PEG8000, aliquoted, and stored at −70 °C.

To obtain high-activity MASP-2, Flag-tagged MASP-2 was expressed in 293T cells, precipitated with anti-FLAG magnetic beads in lysis buffer with 1 mM EDTA at 4 °C for 2 h, eluted with Flag peptide (Sigma), and concentrated with a 10,000 MWCO ultrafiltration tube (Millipore). The concentration of MASP-2 was assessed using a BCA kit (Thermo), SDS-PAGE analysis and immunoblot analysis with purified prokaryotically expressed MASP-2 as a standard control.

### MASP-2 autoactivation and C4 cleavage assay

Purified MASP-2 (8 nM) was incubated at 37 °C for 3 h in 20 mM Tris-HCl (pH 7.4), 150 mM NaCl, and 2 mM CaCl_2_ with purified C4 (Calbiochem), recombinant MBL (Calbiochem), mannan (Sigma), and SARS-CoV N protein with/without C1INH (Calbiochem), anti-MASP-2 (Santa Cruz), and anti-SARS-CoV N antibody (Sino Biological) at concentrations of 50 nM, 30 nM, 15 ng/ml, 10 nM, 40 μg/ml, 2.4 μg/ml and 12 μg/ml, respectively. The cleavage was followed by SDS-PAGE under reducing conditions, and the C4alpha’ fragments, which are left by C4a release after C4 cleavage and can indicate C4 activation, and MASP-2 were detected by an immunoblot analysis with an anti-C4alpha antibody (Santa Cruz) or anti-Flag antibody (Sigma). Purified C4b (Calbiochem) containing C4alpha’, but not C4a, was used to indicate the C4alpha’ band position.

### Complement deposition assay

The C4b deposition assay was performed using a human MBL/MASP-2 assay kit (Hycult Biotech).^63^ In brief, C1q-depleted serum (Calbiochem, 1:100 dilution) or MBL-depleted serum (obtained by immunoprecipitation with an anti-MBL antibody from human serum (HS, Gemini)) was incubated in mannan-coated plates with high salt binding buffer (10 mM Tris-HCl, 10 mM CalCl_2_, 1 M NaCl, and 0.05% Triton X-100, pH 7.4) overnight at 4 °C and removed by washing, and the MBL:MASP-2 complex was captured. Purified C4 in the kit and purified N protein were added and incubated at 37 °C for 1.5 h, and the deposited C4b was detected following standard protocols. The functional activity of LP and AP was analyzed by ELISA as previously described.^32,63^ Nunc Maxisorb plates were coated with 10 μg mannan per well in 100 mM Na_2_CO_3_/NaHCO_3_ (pH 9.6) at room temperature overnight. After each step, the plates were washed three times with TBST-Ca^2+^ (10 mM Tris-HCl, pH 7.4, 140 mM NaCl, 0.05% Tween-20, and 5 mM CaCl_2_). The residual binding sites were blocked by incubation with 10 mM Tris-HCl (pH 7.4), 140 mM NaCl, and 1 mg/ml HSA for 2–3 h at room temperature. Serum samples were diluted in 10 mM Tris-HCl (pH 7.4) containing 150 mM NaCl, 0.5 mM MgCl_2_, 0.05% Tween-20, and 0.1% gelatin with or without 2 mM CaCl_2_ and N protein. All samples and buffers were prepared on ice. Then, the plates were sequentially incubated for 1 h at 4 °C and 1.5 h at 37 °C, followed by washing. All incubation volumes were 100 μl. Complement binding was detected using antibodies followed by washing. The detection of C4, activated C3, and C5b-9 was performed using an anti-C4α chain antibody (Santa Cruz), anti-activated C3 antibody (Santa Cruz), and anti-C5b-9 antibody (Calbiochem), respectively. Antibody binding was detected using an HRP-conjugated sheep anti-mouse antibody or donkey anti-rabbit antibody (R&D). The enzyme activity of HRP was detected using TMB incubation for 30–60 min at RT, and the reaction was stopped with 2 M H_2_SO_4_. The OD was measured at 450 nm using a microplate reader.

### Cell-ELISA

The quantification of complement system activation was performed using a previously described method based on cell-ELISA technology, with modifications.^64^ Briefly, Vero E6 cells were infected with or without SARS-CoV-2 (MOI = 1) or the SARS-CoV-2 S protein-pseudotyped virus (HIV/SARS-CoV-2 S, MOI = 0.2) for 48 h. Then, 20% diluent human serum (HS), anti-MASP-2 antibodies (OMS721 from Omeros prepared by Sino Biological), anti-N antibodies (nCoV396) or IgG4 antibodies (0.5 μg) and SARS-CoV-2 N protein (100 nM, only in HIV/SARS-CoV-2 S infection experiments) were added to each well, and the samples were incubated for 3 h at 37 °C. Then, the cells were washed and fixed with 4% paraformaldehyde for 30 min. Subsequently, each well was washed and blocked with HSA (1 mg/ml) for 1 h, biotin-conjugated anti-MASP-2 antibodies were added, and the deposition of MASP-2 was analyzed by ELISA.

### MASP-2:MBL binding assay

The binding of MASP-2 to MBL was assessed by ELISA. As mentioned above, Nunc Maxisorb plates were coated with 10 μg mannan per well in 100 mM Na_2_CO_3_/NaHCO_3_ (pH 9.6) at room temperature overnight and blocked with 1 mg/ml HSA. MBL protein (1 μg/ml) was incubated in 10 mM Tris-HCl (pH 7.4), 150 mM NaCl, 5 mM CaCl_2_, 100 μg/ml HSA, and 0.5‰ Triton X-100 at 4 °C for 2 h. Purified MASP-2 and N (or control) proteins were added to the wells at different times to obtain final concentrations of 0.2 mg/ml and 200 ng/ml, respectively. The plates were washed after 32 h of incubation at 4 °C, and the binding of MASP-2 was detected with an anti-MASP-2 antibody followed by an HRP-conjugated rabbit anti-goat antibody. The enzyme activity of HRP was detected using TMB incubation for 30–60 min at RT, and the reaction was stopped with 2 M H_2_SO_4_. The OD was measured at 450 nm using a microplate reader.

### Opsonocytophagic assay

The effect of the SARS-CoV N protein on opsonization by the products of complement C3, such as C3b and iC3b, was performed using a previously described method based on the opsonocytophagic assay, with modifications.^65,66^ Briefly, mouse cells isolated from the peritoneal cavity were washed and inoculated with RPMI 1640 media (10% FBS) in 96-well plates for 2 h at 37 °C. The serum was diluted by 0.781, 1.562, 3.125, 6.25, 12.5, 25, 50, and 100% with 1×PBS, 1 mM CaCl_2_, and 2 mM MgCl_2_. The diluted serum, SARS-CoV N protein or HSA (100 ng/ml) and *E. coli* (the ratio to cells was 10:1) were added to each well, and the samples were incubated for 30 min at 37 °C. The reaction was terminated by incubation with 4% paraformaldehyde for 30 min. Complement C3 deposition was detected with a FITC-C3c antibody (Abcam), and the stained cells were counted. The points represent the mean values of two repeated wells. Error bars, mean ± SD. Exact P-values are shown by an unpaired two-tailed Student’s *t*-test between two groups.

### Animal experiments

Groups of BALB/c mice were provided by the experimental animal center of the Academy of Military Medical Sciences. The *Masp2*^*−/−*^ (KO) C57BL/6 N mice (identified by PCR with m*Masp2*-F: 5′-GGTTCTGTCCCCCTGAAATCATTC-3′, m*Masp2*-R: 5′-CACACCTGCTTTCCGCTTACCTTC-3′, and m*Masp2*-R-homo: 5′-GGACTGGGGAGATTCCTCAATGG-3′, and sequencing) and wild-type (WT) littermate mice were provided by Cyagen Biosciences Inc. All mice were maintained in the experimental animal center of the Academy of Military Medical Sciences (China). The mice (8–10 per group, female, 12–15 weeks old) were infected three times (days 1, 2, and 3) with 1 × 10^8–9^ PFU Ad-SARS N/Ad-MERS N/Ad (Beijing BAC Biological Technologies) or a saline control via the tail vein, and LPS (5 mg/kg for the BALB/c mice or 10 mg/kg for the C57BL/6N mice) was given via the tail vein on the 7th day. An anti-MASP-2 monoclonal antibody (200 μg/kg, HBT), anti-N monoclonal antibody (200 μg/kg, Sino Biological) or C1INH (4 mg/kg, Calbiochem) was injected via the tail vein 30 min before LPS injection.

In the pseudoviral infection experiments, the mice (BALB/c, female, 12–15 weeks old, 5–10 per group) were pre-infected with Ad-SARS N/Ad (1 × 10^9^ PFU, days 1, 2, 3) and injected with pseudovirus (1 × 10^7^ PFU, expressing SARS-CoV S or VSV-G, kindly provided by Wenjie Tan, Chinese Center for Disease Control and Prevention, Beijing, China). Seventy-two hours later, an anti-MASP-2 monoclonal antibody (200 μg/kg, HBT), C1INH (4 mg/kg, Calbiochem) or saline was injected via the tail vein immediately before pseudovirus injection. Blood was collected 6 h after pseudovirus injection via the tail vein. Serum LTB4 was detected with an ELISA kit (Cayman).

Here, we used mouse-adapted SARS-CoV-2 (SARS-CoV-2 MA),^67^ a recombinant virus that can use mouse ACE2 for entry into cells. Briefly, WT and Masp2^*−/−*^ (KO) mice (C57BL/6N, female, 12–15 weeks old, 4–5 per group) or mice (BALB/c, female, 12–15 weeks old, 3–4 per group) were infected with 10^5^ PFU intranasally under ketamine–xylazine anesthesia. Seventy-two hours later, the BALB/c mice were treated with an anti-MASP-2 antibody (10 mg/kg, OMS721) or anti-SARS-CoV-2 N antibody (10 mg/kg, nCoV396) via the tail vein. Then, the percentage of lymphocytes (Lym%) and the percentage of neutrophils (Neu%) were analyzed 6 days after infection.

### Immunofluorescence and immunohistochemistry

Postmortem autopsy of 4 patients who died in Huoshenshan Hospital was carried out by Dr. Xiuwu Bian under the approval of the hospital ethics committee and the family members of the patients. Paraformaldehyde-fixed lung tissues were used for paraffin tissue sections and immunohistochemical staining with SARS-CoV-2 N (Sino Biological Inc.), MBL (Santa Cruz), MASP-2 (Santa Cruz), C4α chain (Santa Cruz), C3 (Santa Cruz) or C5b-9 (Calbiochem) antibodies as described in the instruction manual. The in situ PLA of mouse and human lung tissue sections was performed using a Duolink™ In Situ PLA kit (Sigma).

### Detection of C3a and C5a in COVID-19 patients

Sera were collected from mild or critical COVID-19 patients under the approval of the hospital ethics committee. Critical patients who were in the ICU were defined as having fever or suspected respiratory infection plus one respiratory rate >30 breaths/min, severe respiratory distress, or SpO2 < 90% in room air. Patients with pneumonia and no signs of severe pneumonia were defined as mild cases. Sera collected from 10 mild patients, and 18 critical patients were assayed. Sera from 10 healthy people were collected from the clinical laboratory. The serum C3a and C5a levels were detected by double antibody sandwich ELISA (R&D Systems). The serum C3a in some samples was not tested because of insufficient sample volume.

### Statistical analysis

The statistical analyses were performed using GraphPad Prism software version 8.4.0.

### Reporting summary

Further information on research design is available in the Nature Research Reporting Summary linked to this article.

## Supplementary information

Supplementary Materials

Reporting Summary

## Data Availability

All the data shown in this paper are available from the corresponding authors upon reasonable request.
