# Supplementary material for: Highly pathogenic coronavirus N protein aggravates inflammation by MASP-2-mediated lectin complement pathway overactivation
Source: Signal Transduct Target Ther. 2022 Sep 14;7:318. doi: 10.1038/s41392-022-01133-5 (PMC9470675; doi:10.1038/s41392-022-01133-5)
Supplement: Supplementary file 1 — Supplementary Materials [file 41392_2022_1133_MOESM1_ESM.docx]

Supplementary Materials for

Highly pathogenic coronavirus N protein aggravates inflammation by MASP-2-mediated lectin complement pathway over-activation

Ting Gao^1#^, Lin Zhu^1, 2#^, Hainan Liu^1#^, Xiaopeng Zhang^1#^, Tingting Wang^2^, Yangbo Fu^1^, Hongzhen Li^4^, Qincai Dong^1^, Yong Hu^1^, Zhang Zhang^1^, Jing Jin^2^, Zijing Liu^1^, Weihong Yang^2^, Yaoning Liu^2^, Yanwen Jin^1^, Kaitong Li^4^, Yongjiu Xiao^5^, Junli Liu^1^, Huailong Zhao^1^, Yue Liu^1^, Ping Li^1^, Jibo Song^6^, Lu Zhang^5^, Yuwei Gao^7^, Sisi Kang^8^, Shoudeng Chen^8^, Qingjun Ma^1^, Xiuwu Bian^3^, Wei Chen^1^, Xuan Liu^1*^, Qing Mao^3*^, and Cheng Cao^1*^

^#^Co-first authors:

These authors contributed equally to this study.

^*^Correspondence to:

Cheng Cao (caoc@nic.bmi.ac.cn), Qing Mao ([qingmao@tmmu.edu.cn](mailto:qingmao@tmmu.edu.cn)) or Xuan Liu ([liux931932@163.com](mailto:liux931932@163.com))

**This PDF file includes:**

Supplementary Figures 1 to 5


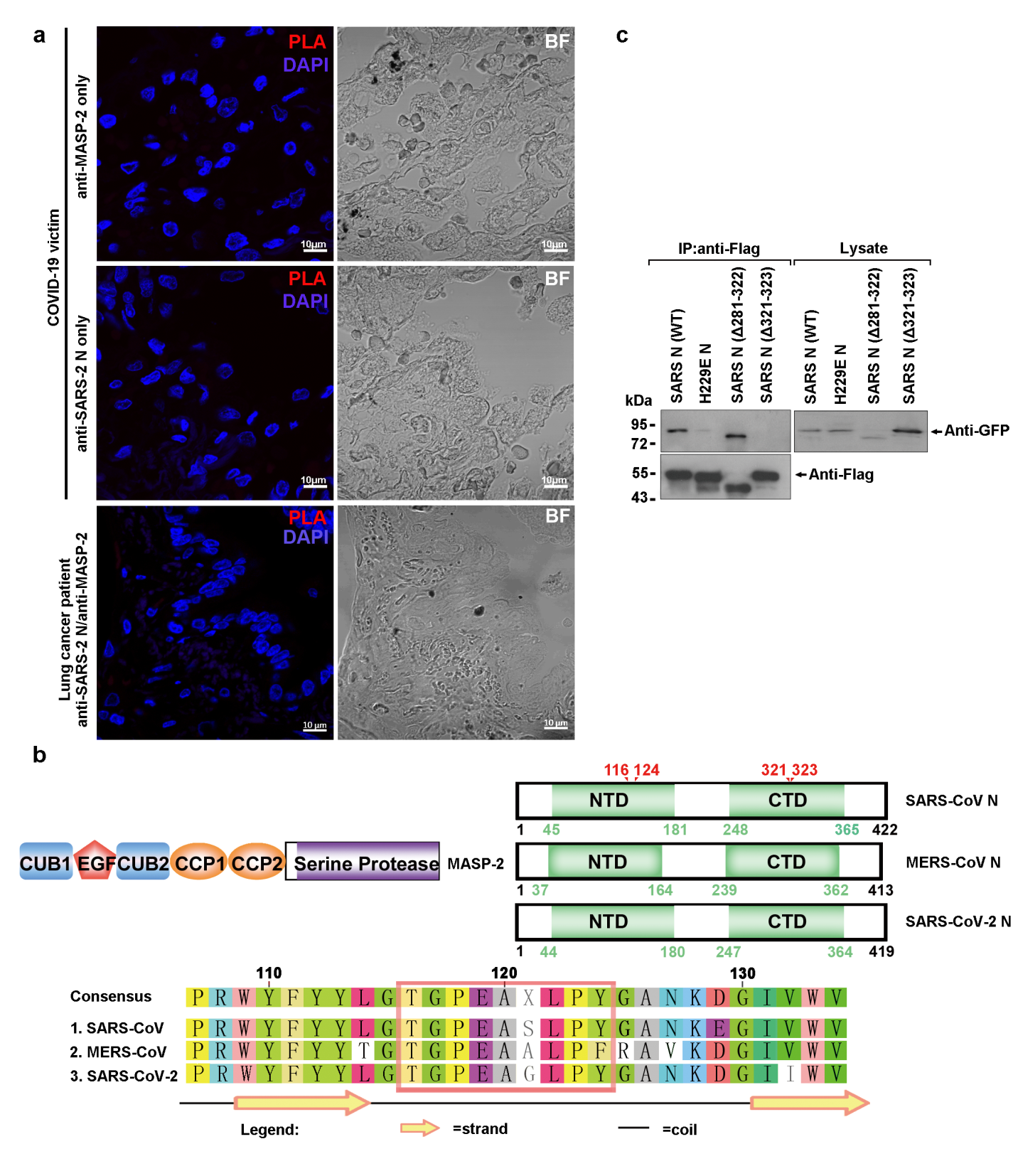
Supplementary Fig. 1 Binding of coronavirus N protein and human MASP-2. a Paraformaldehyde-fixed lung tissues from COVID-19 patients (upper panel) or tumor resection (lower panel) were used for paraffin tissue sections. SARS-CoV-2 N:MASP-2 complex formation in the lung was determined by *in situ* PLA with (lower panel) or without (upper panel and middle panel) anti-SARS-CoV-2 N and anti-MASP-2 antibodies as indicated by the red signals. Scale bar, 10 μm. b Domains of MASP-2 and motif of highly pathogenic human coronavirus N protein involved in the association. c Lysates of 293T cells expressing Flag-tagged N protein or mutants were incubated with anti-Flag agarose beads, and the N-conjugated agarose beads normalized for the N protein level were incubated with lysates of 293T cells expressing GFP-tagged N proteins and mutants. The adsorbates were analyzed by immunoblotting with anti-GFP.

**
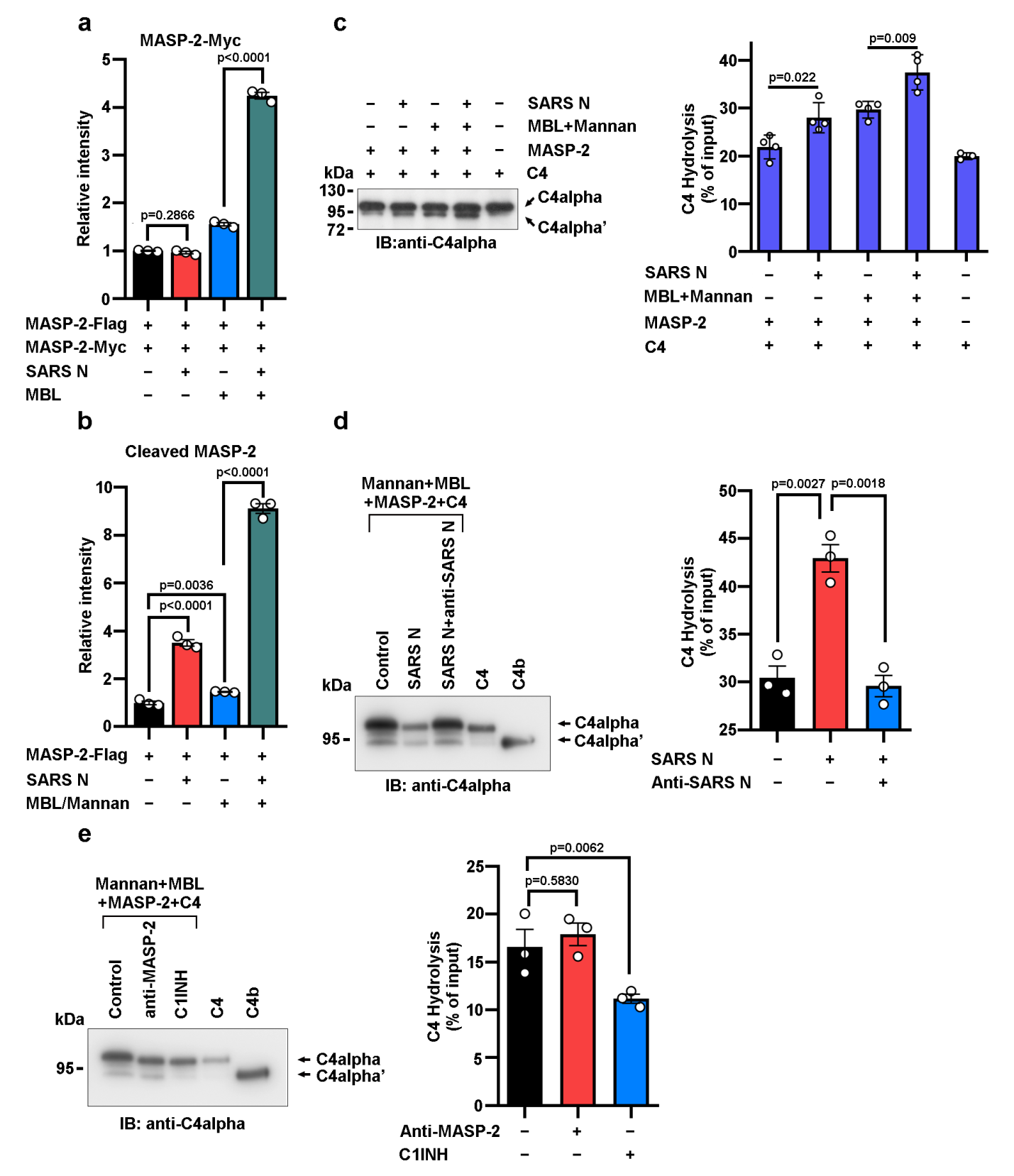
Supplementary Fig. 2** N proteins of SARS-CoV and MERS-CoV potentiate MASP-2 dimerization, cleavage, and C4 cleavage. **a, b** MASP-2-Myc in Fig. 3a **(a)** and Cleaved MASP-2 in Fig. 3b **(b)** were quantified by densitometry with ImageJ software (n=3). The differences between the two groups were evaluated using a two-sided unpaired Student’s t-test. The data are presented as the mean±SEM. **c-e** C4 was incubated with/without MASP-2, MBL, mannan or SARS-CoV N protein **(c)**, an anti-SARS-CoV N antibody **(d)**, C1INH or an anti-MASP-2 antibody **(e)** at 37°C for 2 hr. C4 and a cleaved truncated C4 fragment were detected with an anti-C4alpha antibody (left panel). The C4 cleavage rate was calculated using the formula truncated C4/ (truncated C4+remaining C4) ×100% by densitometric analysis with ImageJ software (n=3) (right panel). The data are presented as the mean±SEM.


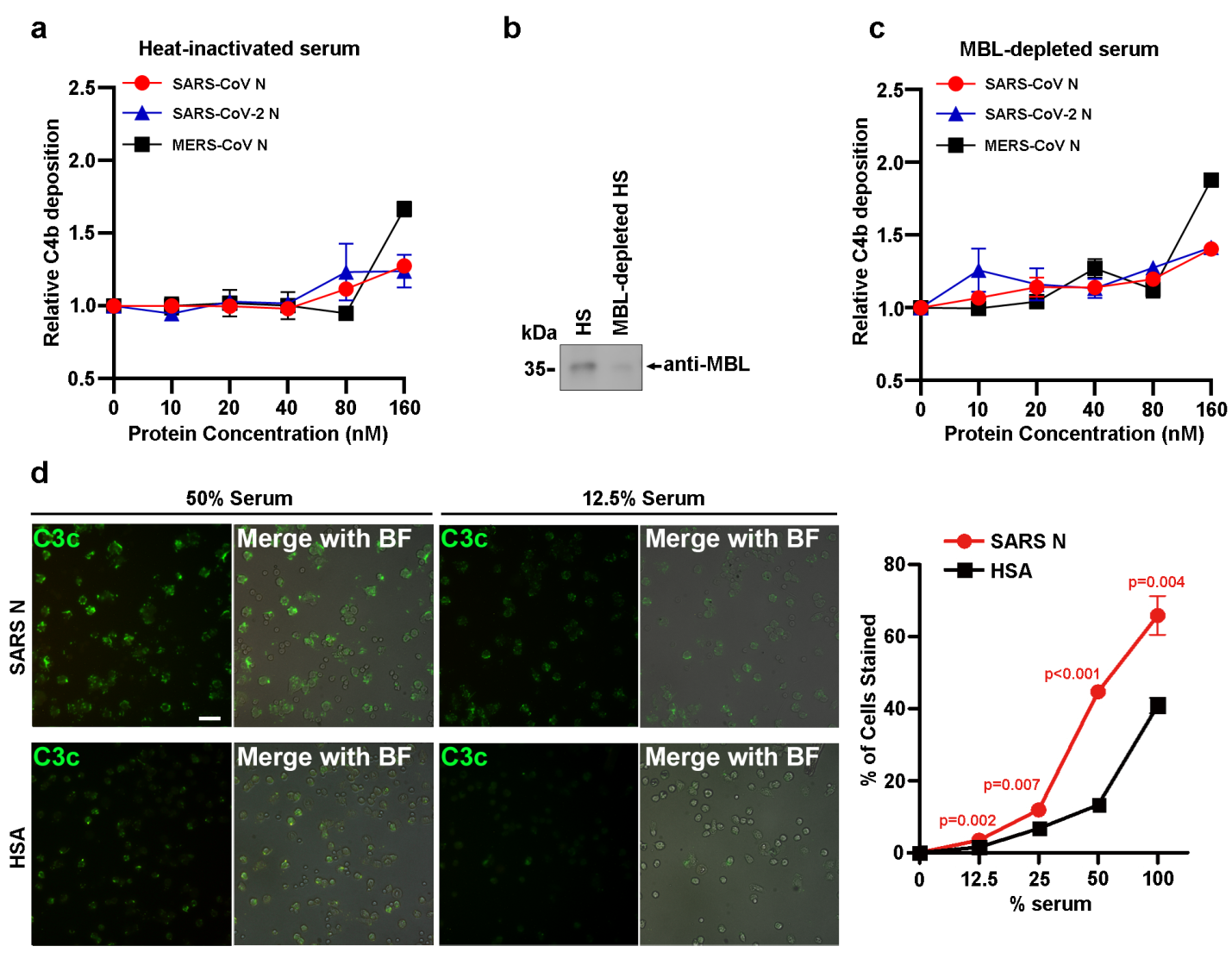


**Supplementary Fig. 3** N proteins of SARS-CoV, MERS-CoV and SARS-CoV-2 accelerate the lectin complement pathway cascade. **a, c** The effect of MERS-CoV, SARS-CoV and SARS-CoV-2 N protein on C4b deposition in heat-inactivated serum **(a)** and MBL-depleted serum **(c)** was determined. The OD450 value of N protein at 0 nM was set to 1 to calibrate the relative C4b deposition level. The data are presented as the mean ±SEM of two tests. The statistical analysis was performed using an unpaired two-tailed Student’s *t*-test. **b** MBL-depleted serum diluted 20-fold was analyzed by immunoblotting with the indicated antibodies. **d** Opsonocytophagic test of mouse macrophages in serum in the presence or absence of SARS-CoV N protein. HSA was used as a negative control. The deposited C3 fragment was stained with a FITC-tagged C3c antibody (binding to C3b and iC3b). Microphotography was carried out by a fluorescence microscope, and some photos are shown (left panel). Scale bar, 50 μm. The points represent the mean values of two repeated experiments (right panel). Error bars, mean±SD. The statistical analysis was performed using an unpaired two-tailed Student’s *t*-test.

**
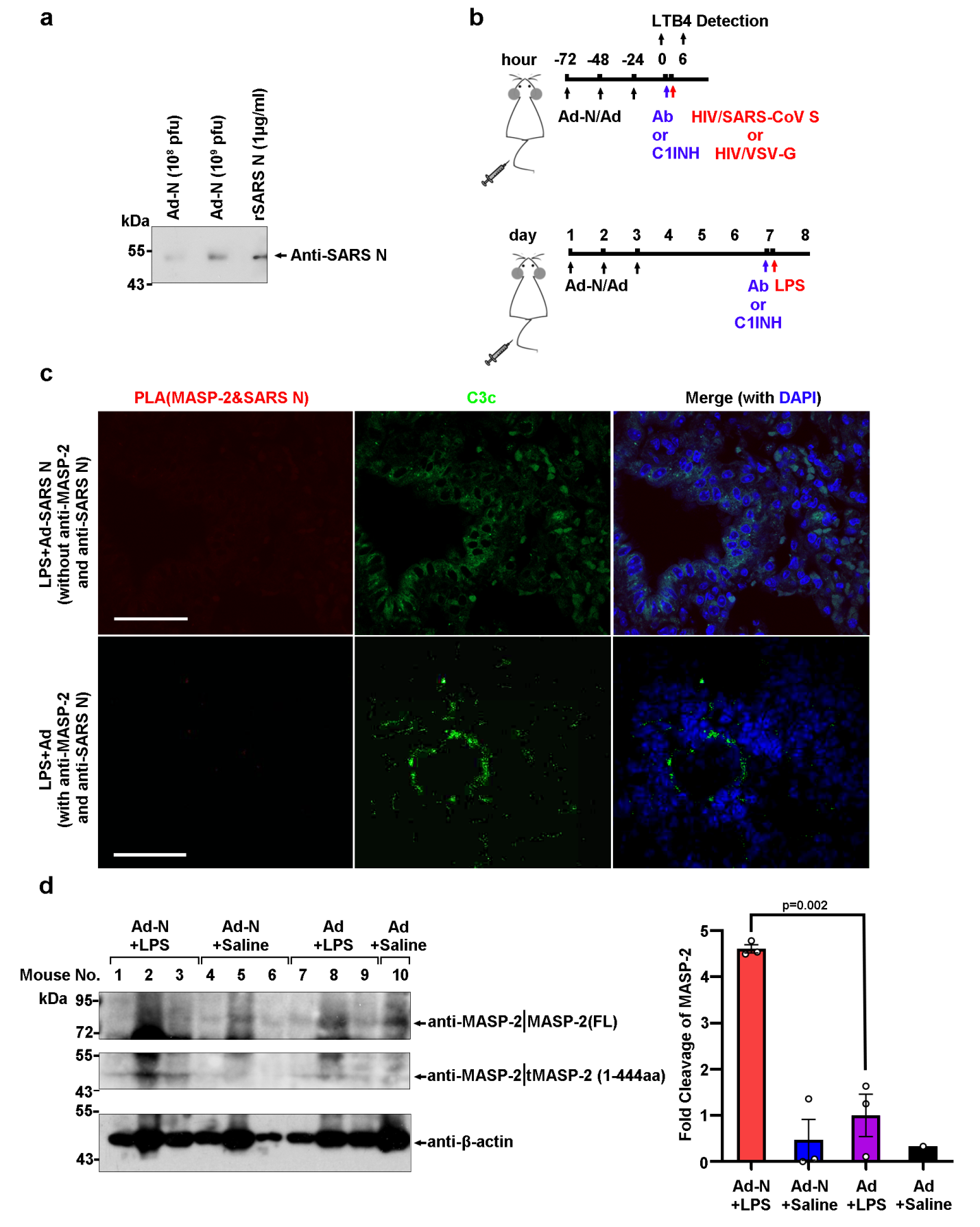
Supplementary Fig. 4** The SARS-CoV N protein accelerates the lectin complement pathway cascade *in vivo*. **a** Mice were infected with Ad-SARS N (1×10^8^ PFU), and the serum N protein was detected with an anti-N antibody. Recombinant N protein was used as a standard. **b** A diagram of the Ad-N pre-infected and S-pseudotyped HIV/LPS challenged mouse model. **c** Mice were infected with 1×10^8^ PFU Ad-SARS N (upper panel) or Ad (lower panel), and LPS was injected on the 7^th^ day post-infection. The mice were sacrificed 6 hr after the LPS challenge. SARS-CoV N and MASP-2 complex formation in frozen lung sections was measured by *in situ* PLA with (lower panel) or without (upper panel) anti-SARS-CoV N and anti-MASP-2 antibodies. Deposited C3 fragments were stained with a FITC-labeled anti-C3c antibody (green). Scale bar, 50 μm. **d** Mice were infected with 1×10^8^ PFU Ad-SARS N or Ad, and LPS was administered on the 7^th^ day. The mice were sacrificed 6 hr after the LPS injection. Lung tissue homogenate was subjected to WB with anti-MASP-2 and anti-β actin antibodies (left panel). The MASP-2 cleavage rate was calculated using the formula truncated MASP-2/ (truncated MASP-2+remaining MASP-2) ×100% by a densitometric analysis (right panel).


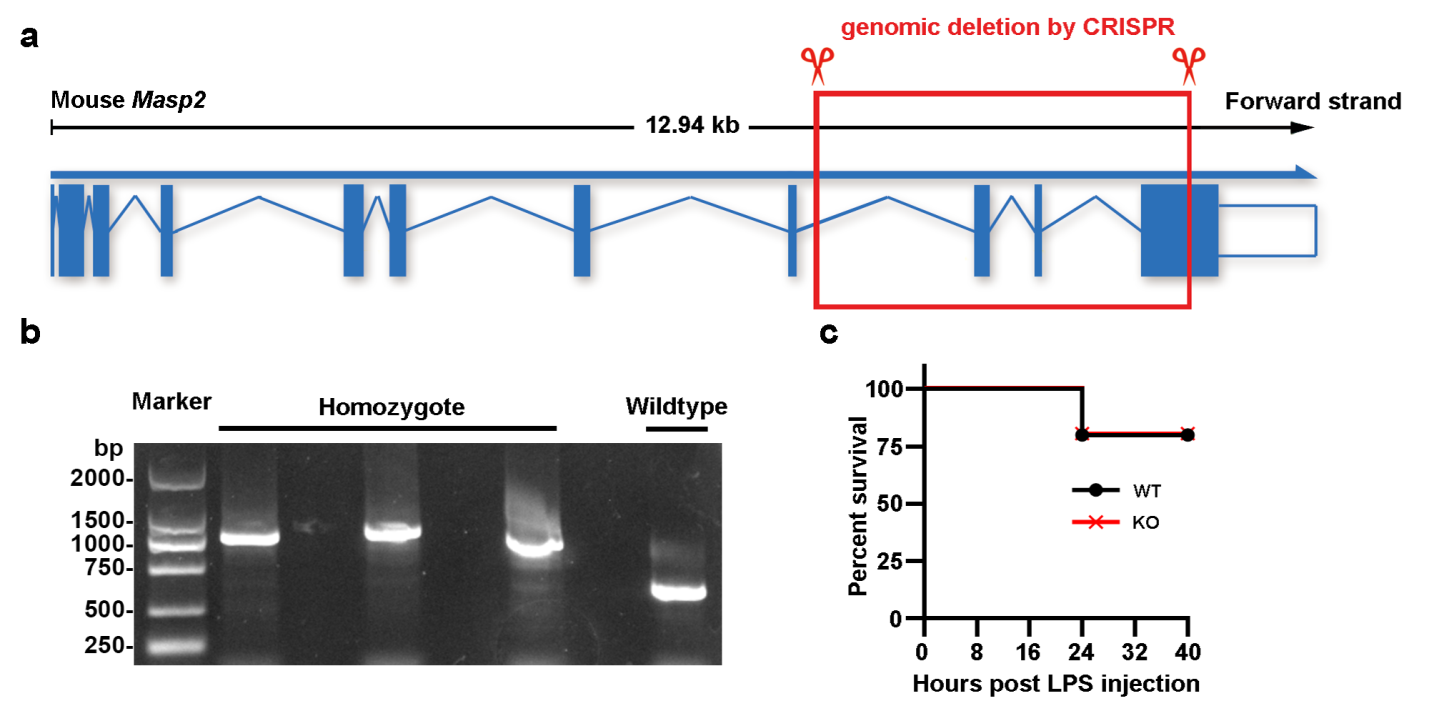


Supplementary Fig. 5 *Masp2* knockout mice. a A diagram of the mouse *Masp2* gene knockout. To create the *Masp2* knockout mouse model (C57BL/6N) by CRISPR/Cas-mediated genome engineering, exon 9 to exon 11 of the mouse *Masp2* gene were selected as target sites. b DNA from mouse tails was identified by PCR. c As described in Fig. 6d, WT and *Masp2^-/-^* mice without adenovirus preinfection were challenged with LPS, and the survival curves were plotted.
